# Effect of Mn Content on the Microstructure, Mechanical Properties, and Damping Capacity of Mn-Cu Alloys

**DOI:** 10.3390/ma19091742

**Published:** 2026-04-24

**Authors:** Bin Wu, Bibo Li, Zhaobo Wu, Fengshuang Lu, Ran Li, Xiaojun Zhang, Xinqing Zhao, Feiyu Zhao, Dongliang Zhao

**Affiliations:** 1 Central Iron and Steel Research Institute, Beijing 100081, China; w2825802@163.com (B.W.); wuzhaobo@nercast.com (Z.W.); lufengshuang@163.com (F.L.); liran@cisri.cn (R.L.); zxjun1183@163.com (X.Z.); zfy_tom@sina.com (F.Z.); 2 School of Materials Science and Engineering, Beihang University, Beijing 100191, China; xinqing@buaa.edu.cn; 3 China Iron & Steel Research Institute Group Co., Ltd., Beijing 100081, China

**Keywords:** Mn content, Mn-Cu alloys, fcc-fct phase, twin boundaries, damping capacity

## Abstract

This study investigated the influence of Mn content (70 wt.%, 75 wt.%, and 80 wt.%) on the microstructure, mechanical properties and damping capacity of Mn-Cu alloys using X-ray diffraction (XRD), scanning electron microscopy (SEM), transmission electron microscopy (TEM), mechanical testing and dynamic mechanical analysis (DMA). The results indicate that during cooling after aging, the Mn-Cu alloy undergoes martensitic transformation, resulting in a dual-phase structure of fcc and fct. The 70 wt.% Mn alloy exhibits a mixed-grain structure with mostly long, straight twin bands, while the 75 wt.% and 80 wt.% Mn alloys consist of fine equiaxed grains with mostly intersecting twin bands. The microstructure determines the properties of the alloy. As the Mn content increases, the mechanical properties initially increase and then decrease, and the 75 wt.% Mn alloy has the best mechanical performance (UTS = 534 MPa, YS = 263 MPa). In contrast, the damping capacity shows a decreasing trend, and the 70 wt.% Mn alloy exhibits the best damping capacity (tanδ = 0.064). The main damping peak of tanδ in Mn-Cu alloys is derived from the relaxation of the twin boundaries, and the less obvious secondary peak is the internal friction peak of martensitic transformation.

## 1. Introduction

The damping capacity of Mn-Cu alloys exhibits considerable potential for vibration and noise attenuation applications, such as in precision instruments, aerospace, and mechanical engineering, owing to their remarkable damping performance, good mechanical properties and corrosion resistance [1,2,3]. The damping capacity of Mn-Cu alloys is primarily attributed to reversible martensitic transformation or stress-induced twinning [4,5,6]. Chemical composition, especially Mn content, plays a significant role in controlling the phase transformation behavior, microstructural evolution and damping capacity [7,8,9]. For instance, alloys containing more than 69 at.% Mn have an extremely high damping capacity, even without any heat treatment [9,10]. However, alloys with excessively high Mn content tend to exhibit poor workability under both cold and hot working conditions. Aging heat treatment, pre-deformation before aging heat treatment [11,12], cryogenic treatment [13], lowering the Mn content and adding other suitable quantities of elements can improve better processability and synergistic strengthening of these alloys [14,15,16]. In contrast, Mn-Cu alloys with lower Mn content require aging heat treatment within an optimum temperature to obtain superior damping characteristics [12,17,18], indicating that the microstructure is also an important factor that affects damping behavior. Tsuchiya et al. [18] explored the effect of isothermal aging on the fcc-fct martensitic transformation behavior of Mn-14 at.% Cu alloy. They discovered that the non-uniform structure resulting from spinodal decomposition during the aging treatment is responsible for the increase in martensitic transformation temperature. Liu et al. [19] characterized the microstructure, mechanical properties, and damping capacity of Mn63.6Cu26 alloy fabricated by Laser Powder Bed Fusion (LPBF) process. The findings indicate that LPBF followed by heat treatment after fabrication produces excellent tensile strength and damping capacity in Mn-Cu alloys. Li et al. [20] showed that neither spinodal decomposition nor twinning is induced by solution treatment in Mn-Cu alloys. The ensuing aging treatment, however, induces spinodal decomposition and the formation of Mn-rich regions, which raises the martensite start temperature (Ms). This enhances the martensitic transformation and the formation of beneficial twin boundaries, thus further improving both damping capacity and mechanical properties. Zuo et al. [21] investigated the internal friction of a Mn-11 at.% Cu alloy by applying tensile stress along the [001], [011] and [111] directions. They found that the damping peak between −73 °C and 27 °C is caused by twin boundary relaxation. The highest damping occurs in the [001] direction, and the lowest damping occurs in the [111] direction, depending on the Schmid factor of {110} twin boundaries in each orientation. The peak of the reverse martensitic transformation occurs at approximately 157 °C with little orientation dependence.

Very few studies have focused on the Mn content, and most have only considered its influence on the damping performance. Most current research has focused on the effects of heat treatment parameters and alloying elements on Mn-Cu alloys with a single Mn content, whereas studies on alloys with varying Mn contents are sparse. Nevertheless, systematic studies on the interdependence among Mn content, phase constitution, specific microstructural features (grain size, precipitates), mechanical properties (strength and ductility), and damping capacity are still needed. Thus, this study aims to systematically investigate several Mn-Cu alloys with varying Mn contents. Accordingly, the present study aims to elucidate the influence of Mn content on the microstructure, mechanical properties, and damping capacity of Mn-Cu alloys, and provide guidance for the application-oriented selection of Mn content. The results are expected to provide a theoretical foundation and experimental evidence for optimizing the composition design of Mn-Cu alloys and enhancing their performance in engineering applications.

## 2. Materials and Methods

Mn-Cu alloys with increasing nominal Mn contents (70, 75, and 80 wt.%) were prepared, with the concentrations of the other elements reduced proportionally, to investigate the effect of Mn content on the microstructure and properties. The alloys were prepared by vacuum induction melting using electrolytic manganese blocks (purity > 99.9%), electrolytic copper blocks (purity > 99.9%), carbonyl nickel blocks (purity > 99.5%) and electrical pure iron blocks (purity > 99.5%) as raw materials. The raw materials were weighed according to the target compositions and placed in a vacuum induction furnace (ZGJL0025-100-25BG, Jinzhou Transformer and Electric Furnace Factory, Jinzhou, China) sequentially. The vacuum was better than 5 × 10^−3^ Pa, and the furnace was filled with 40 kPa argon until all the raw materials were melted. After that, the molten raw materials were fully stirred to ensure uniform composition. After removing the oxide skin and riser, the ingot was homogenized at 880 °C for 2 h and then hot-rolled into plates. The plates were solution-treated at 880 °C for 1 h, water-quenched, and then aged at 425 °C for 4 h. The chemical compositions of the prepared alloys were determined by an inductively coupled plasma emission spectrometer (ICP-OES, iCAP6300 Radial, Thermo, Waltham, MA, USA) on selected samples, and the average of three duplicate measurements was taken as the final result, as shown in Table 1. The manganese contents of 70%, 75% and 80% were the nominal components in this study. Owing to losses during the smelting process, slight deviation existed between the actual and the nominal components.

To fully characterize the microstructure, mechanical properties and damping capacity, various analytical techniques were used. The samples were subjected to conventional metallographic preparation (mechanical grinding and polishing) followed by phase analysis with a Cu-Kα X-ray diffractometer (XRD, DX-2700B, Malvern Panalytical-Empyrean, Worcestershire, UK). The metallographic samples were polished and stress-relieved using an ion polishing instrument (RES 101, Leica, Wetzlar, Germany) to reveal the microstructure. Microstructural features were observed using a field-emission scanning electron microscope (SEM, Apreo 2C, Thermo, USA), equipped with an electron backscatter diffraction (EBSD, Oxford Instruments, Abingdon, UK) detector for texture analysis and an energy-dispersive X-ray spectrometer (EDS, Oxford Instruments, UK) for localized compositional analysis. The accelerating voltage of the scanning electron microscope was 20 kV, the working distance was 10 mm, and the EBSD step size was 0.5 µm. The samples were cut into 10 mm × 10 mm × 0.3 mm flakes and ground to a thickness of 50 μm using silicon carbide sandpaper, and finally punched into 3 mm diameter wafers. Then, the wafers were milled using an ion thinning instrument (Gatan 695, Gatan, Pleasanton, CA, USA). The initial thinning energy was 5 keV, and the thinning angle was 10°. After perforation, the thinning energy was reduced to 3.5 keV and the angle to 5°. Subsequently, the microstructure of the samples was further observed by 200 KV transmission electron microscopy (TEM, H800, Hitachi, Tokyo, Japan).

Mechanical properties were evaluated using a universal materials testing machine (Z100, Zwick/Roell, Ulm, Germany) at a tensile rate of 2 mm/min, in accordance with the national standard GB/T 228.1-2021 [22]. Three parallel specimens were tested for each alloy condition, and the average values were reported as the final results. The thermal expansion properties of the alloys as a function of temperature were measured using a linear dilatometer (DIL 402 Expedis Select, NETZSCH, Waldkraiburg, Germany) to study the coefficient of physical expansion changes in the alloy over the temperature range from −150 °C to 200 °C. The damping behavior was investigated using a dynamic mechanical analyzer (DMA 850, TA Instruments, New Castle, DE, USA) operating in three-point bending mode. Specimens with dimensions of 50 mm × 10 mm × 2 mm were tested over a temperature range from −100 °C to 200 °C at a heating rate of 5 °C/min and a frequency of 10 Hz. The damping capacity was characterized by the loss tangent (tan δ).

## 3. Results

### 3.1. Effect of Mn Content on Phase Constitution and Microstructure

**Figure 1:**
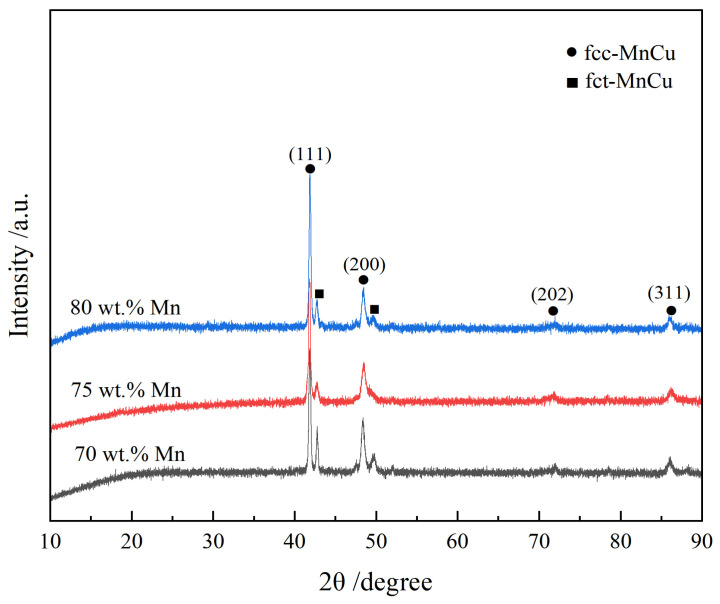
XRD patterns of Mn-Cu alloys with different Mn contents.

Figure 1 presents the XRD patterns of the alloys with different Mn contents after solution treatment at 880 °C for 1 h (water-quenched) followed by aging at 425 °C for 4 h. As illustrated in Figure 1, new diffraction peaks appearing beside the (111) and (200) peaks of the fcc-MnCu phase, corresponding to the fct-MnCu phase. The results show that martensitic transformation occurs in all alloys during cooling, and the alloys are composed of fcc and fct phases after solution and aging treatment.

**Figure 2:**
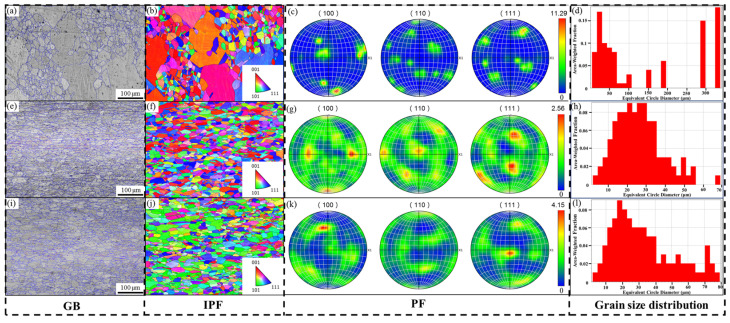
GB, IPF, PF and grain size distribution maps of Mn-Cu alloys with different Mn contents. (**a**–**d**) 70 wt.% Mn; (**e**–**h**) 75 wt.% Mn; (**i**–**l**) 80 wt.% Mn.

The grain boundaries (GB), inverse pole figures (IPF), pole figures (PF) and grain size distribution maps of the Mn-Cu alloys are presented in Figure 2. The grain size of each alloy was calculated using the area-weighted equivalent circular diameter method from EBSD data, and the grains involved in the calculation were intact grains with a misorientation greater than 10°. Figure 2a,d show that after solution and aging treatment, the 70 wt.% Mn alloy exhibits a mixed grain structure consisting of large grains with a maximum size of 322 μm and small grains with a minimum size of 4 μm, resulting in an average area-weighted grain size of 140 μm. The alloys with 75 wt.% Mn and 80 wt.% Mn are composed of fine equiaxed grains with similar grain size distributions, and their area-weighted average grain sizes are 26 μm and 33 μm, respectively, as shown in Figure 2e,h,i,l. The inverse pole figures (IPF) and pole figures (PF) reveal that no strong texture is present in the alloys regardless of Mn content, although the orientation varies slightly. The grains in the 70 wt.% Mn alloy are mainly oriented along the [001] direction with a pole density of 11.29, mainly because the large grains are oriented close to [001]. The grains in the 75 wt.% Mn alloy are mostly oriented along the [001] and [111] directions with a pole density of 2.56. In contrast, the 80 wt.% Mn alloy exhibits a weak [111] texture component with a pole density of 4.15. It has been reported [21] that grains oriented along the [001] direction have the most positive effect on damping properties, followed by the [110] direction and finally the [111] direction. Based on the orientation alone, the 70 wt.% Mn and 75 wt.% Mn alloys may exhibit better damping capacity than the 80 wt.% Mn alloy.

**Figure 3:**
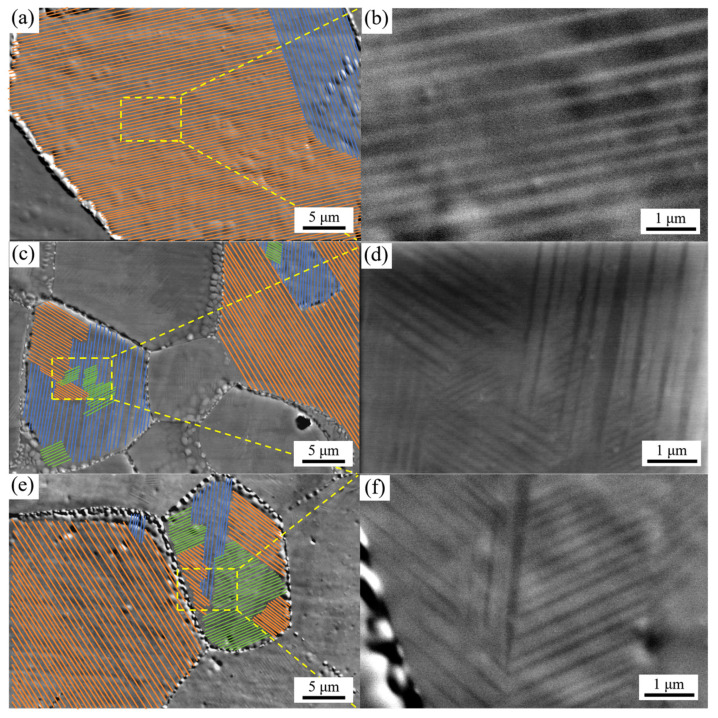
Twin structures within the grains of Mn-Cu alloys with different Mn contents. (**a**,**b**) 70 wt.% Mn; (**c**,**d**) 75 wt.% Mn; (**e**,**f**) 80 wt.% Mn.

High-magnification SEM imaging (Figure 3) revealed that most grains contain at least two sets of twin bands, forming spatially intersecting structures. The intersection of different twin band variants creates junction regions where the twin bands become sharper. In the large grains of the 70 wt% Mn alloy, the micro-twins are arranged mainly in one direction leading to long, straight and uninterrupted twin bands (Figure 3a,b). Conversely, in the smaller grains of the 75 and 80 wt.% Mn alloys, more than two sets of twin bands are typically observed, leading to a greater number of intersecting spatial configurations (Figure 3c–f). Research [23] indicated that twin formation is not only related to aging treatment but also depends on the grain size of the parent fcc phase. Larger grains are more prone to forming a higher density of twin structures after aging, which is consistent with the observations in Figure 3. The constrained twin bands located within the junction regions may reduce the alloy’s damping capacity by hindering the reversible movement of twin boundaries [24,25,26]. Therefore, appropriately increasing the grain size, controlling twin band intersections, and reducing the presence of constrained twin bands could be effective strategies for improving damping performance.

**Figure 4:**
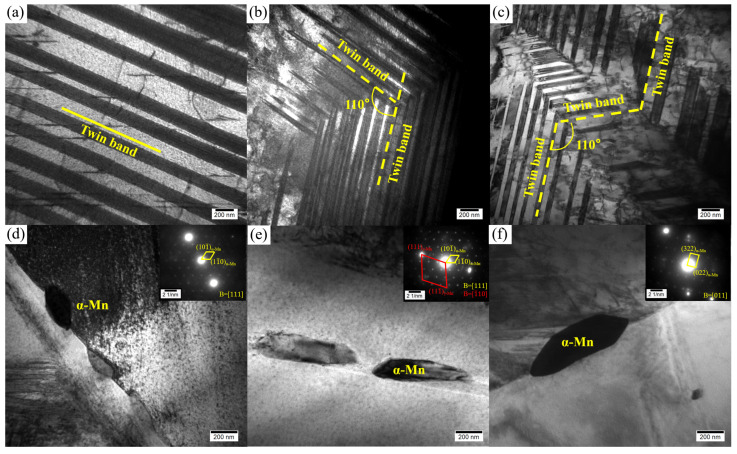
Twins and α-Mn phases in Mn-Cu alloys. (**a**,**d**) 70 wt.% Mn; (**b**,**e**) 75 wt.% Mn; (**c**,**f**) 80 wt.% Mn.

In order to further observe the twin structures and the second phase particles, the Mn-Cu alloys with different Mn contents were analyzed by TEM, and the results are shown in Figure 4. Figure 4a–c show that the twin width in the 75 wt.% and 80 wt.% Mn alloys is smaller than that in the 70 wt.% Mn alloy. Consistent with the results observed in SEM, most of the twins in the 70 wt.% Mn alloy are long and straight, with few cross structures observed. Many groups of twin bands and more intersecting twin structures are present in the grains of the 75 wt.% and 80 wt.% Mn alloys. Additionally, a small number of irregular blocky secondary phase particles were observed at the grain boundaries in all three alloys (Figure 4d–f). The secondary particle size increased with the increasing Mn content. As calibrated by SAED, the particles at the grain boundaries were identified as the α-Mn phase. Nevertheless, their quantity was insufficient to produce detectable diffraction peaks.

### 3.2. Effect of Mn Content on Mechanical Properties

**Figure 5:**
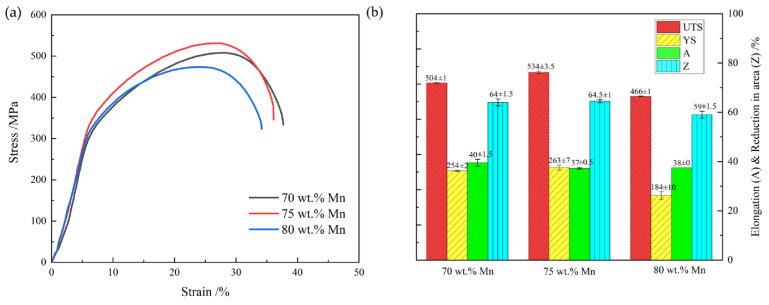
Engineering stress–strain curves (**a**) and corresponding data (**b**) of Mn-Cu alloys with different Mn contents.

The engineering stress–strain curves and the associated data are presented in Figure 5. Mechanical properties show a systematic variation with increasing Mn content. When the Mn content is increased from 70 wt.% to 75 wt.%, the yield strength (YS) increases from 254 MPa to 263 MPa, and the ultimate tensile strength (UTS) increases from 504 MPa to 534 MPa, whereas the elongation (A) decreases slightly from 40% to 37%. With a further increase in Mn content to 80 wt.%, the YS drops abruptly to 184 MPa and the UTS to 466 MPa, and there is a small rise in elongation. This indicates that in the range of investigation Mn content, the strength of Mn-Cu alloys initially grows and then falls with increasing Mn content, and the plasticity has a weak tendency to decrease and then recover slightly. In addition, the reduction in area (Z) has a tendency like that of strength. The 70 wt.% Mn alloy has a reduction in area of 64%, which is slightly increased to 65% in the 75 wt.% Mn alloy, but subsequently goes down to 59% in the 80 wt. % Mn alloy. This further supports the influence of Mn content on plasticity. The 75 wt.% Mn alloy has high plasticity and it possesses the maximum strength, an aspect that is closely related to the finer and more uniform structure.

The observed tendency of strength first increasing and then decreasing is mainly attributed to microstructural evolution. As described in Section 3.1, increasing the Mn component initially refines the grain size, followed by slight coarsening: the 70 wt.% Mn alloy has a mixed-grain morphology where the average area-weighted grain size is about 144 μm; the 75 wt.% Mn alloy has the finest grains, averaging about 26 μm; and the 80 wt.% Mn alloy shows slightly coarsening, with an area-weighted average grain size of approximately 32 μm. According to the Hall–Petch relationship, grain refinement contributes greatly to material strength. Furthermore, with increasing Mn content and decreasing grain size, the micro-twin structures transform from straight, continuous bands dominating large grains to multiple intersecting twin bands in smaller grains (Figure 3). This network-shaped micro-twin structure can efficiently hinder dislocation movement, thus increasing strength (Figure 4a–c). Simultaneously, the small grain size helps maintain plasticity without significant reduction.

The 80 wt.% Mn alloy has a somewhat bigger grain size than the 75 wt.% Mn alloy as an opposing factor. In addition, the increase in Mn content necessarily reduces the concentrations of the other elements in the alloy, while the α-Mn particles on the grain boundaries precipitate and grow in the 80 wt.% Mn alloy, which consumes Mn in the grain and further weakens the solid-solution strengthening effect. Thus, its strength is relatively lower. The decrease in plasticity with increasing Mn concentration may be related to the increased precipitation of the α-Mn particles at grain boundaries, which can also serve as preferential crack initiation sites.

**Figure 6:**
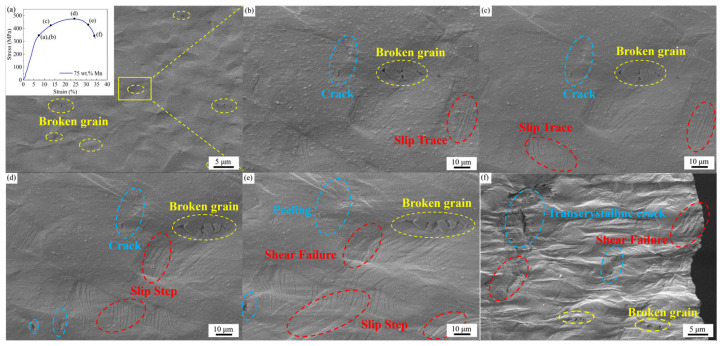
In situ microstructure diagram of MnCu alloy during the tensile process. (**a**,**b**) Initial stage of strengthening; (**c**) late stage of strengthening; (**d**) maximum force point; (**e**) necking stage; (**f**) cross-sectional fracture.

Tensile behavior was investigated using in situ SEM on the Mn-Cu alloys to acquire further insight into the deformation mechanisms. No significant difference was observed among the three alloys in the in situ tensile process, therefore the 75 wt.% Mn alloy with the best mechanical performance was used as a representative for description, as shown in Figure 6. Upon entering the strain-hardening stage, discrete, unidirectional slip bands appear in some of the smaller grains (Figure 6a,b). These slip bands extend to grain boundaries, causing stress concentration at the intersection points and initiate microcracks as the stress rises. At higher tensile stresses, the small grains that have undergone cracking continue to fracture, and slip traces eventually become visible in most grains (Figure 6c,d). With maximal tensile stress applied, necking starts and several families of slip traces occur within the grains, and this triggers shear failure and the formation of transgranular cracks (Figure 6e). Combined with the fragmentation of small grains, this causes material failure (Figure 6f).

**Figure 7:**
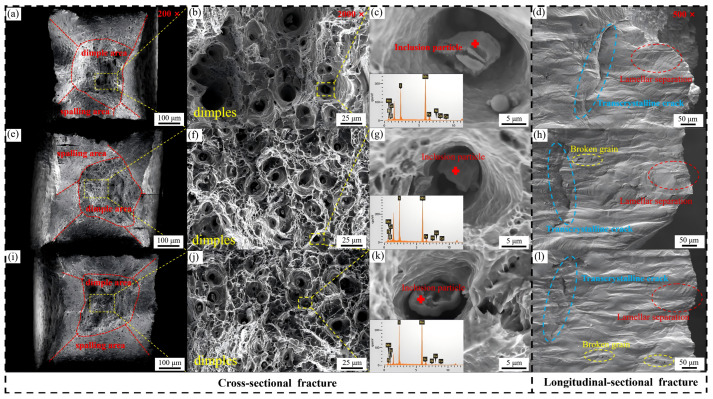
Cross-sectional and Longitudinal-sectional fracture morphologies of Mn-Cu alloys with different Mn contents. (**a**–**d**) For 70 wt.% Mn; (**e**–**h**) for 75 wt.% Mn; (**i**–**l**) for 80 wt.% Mn.

The fracture mechanisms were further elucidated by studying the tensile fracture surfaces (Figure 7). All the alloys exhibit typical features of ductile fracture, consisting mainly of a central dimple region and outer regions with evidence of shear lips, along with transgranular fracture characteristics. In the 70 wt.% Mn alloy, the distribution of dimple sizes is not uniform due to its mixed-grain structure. In the 75 wt.% Mn alloy, the fracture surface shows some small and homogeneous distributed dimples, indicating a remarkable balance between strength and ductility. Conversely, the fracture surface of the 80 wt.% Mn alloy reveals fewer dimples, which are larger and more non-uniformly distributed, with some regions exhibiting tearing ridges. This feature may be attributed to a higher volume fraction of brittle α-Mn particles, consistent with the observed reductions in both elongation and area reduction. Also, a few large particles found on fracture surfaces were identified as fragments from grain disintegration.

### 3.3. Effect of Mn Content on Damping Capacity

**Figure 8:**
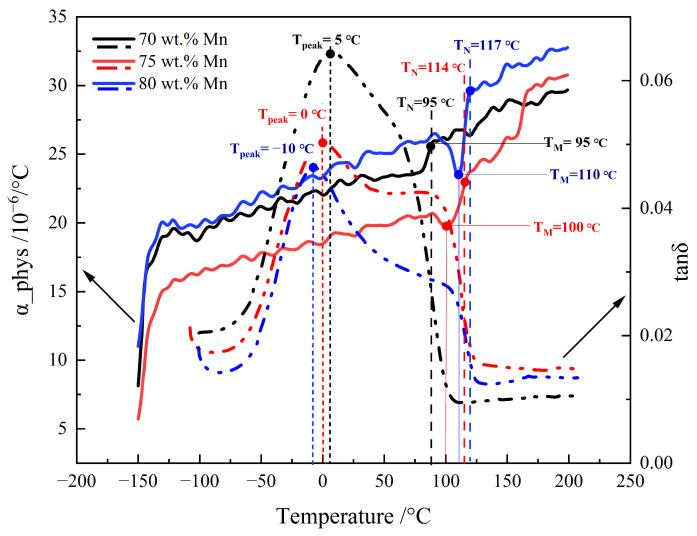
Temperature dependence of the physical expansion coefficient (imaginal line) and loss tangent tanδ (solid line) for Mn-Cu alloys with varying Mn content.

Figure 8 shows the curves of the physical coefficient of linear expansion (α_phys) and loss tangent (tanδ measured at 10 Hz) with regard to temperature dependence. The α_phys of the 70 wt.% Mn alloy shows a slight rise between 80 °C and 95 °C. For the 75 wt.% Mn alloy, α_phys decreases from 90 °C to 100 °C, and then rises sharply before stabilizing at 114 °C. The α_phys of the 80 wt.% Mn alloy shows the same trend as that of the 75 wt.% Mn alloy. The α_phys of 80 wt.% Mn alloy decreased from 100 °C to 110 °C, and then returned to a plateau around 117 °C. The anomalies in α _phys are usually associated with phase transformations, often martensitic or paramagnetic transition in MnCu alloys. The martensitic transformation reduces the volume of the alloy, resulting in a sharp decrease in α_phys. On the contrary, the paramagnetic transition produces a magnetostrictive effect, which makes the alloy expand and α_phys increase [27,28]. In this work, the completion temperature of martensitic transformation is defined as T_M_, and the completion temperature of paramagnetic transition is defined as T_N_. Therefore, in the 75 wt.% Mn alloy, the martensitic transformation occurred between 90 °C and 100 °C (T_M_ = 100 °C), and the paramagnetic transition occurred between 100 °C and 114 °C (T_N_ = 114 °C). The martensitic transformation of 80 wt.% Mn alloy occurred between 100 °C and 110 °C (T_M_ = 110 °C), and the paramagnetic transition occurred between 110 °C and 117 °C (T_N_ = 117 °C). In contrast, the α_phys of 70 wt.% Mn alloy only shows a weak increase between 80 °C and 95 °C, which is caused by the coupling of martensitic transformation and paramagnetic transition. This indicates that both martensitic transformation and paramagnetic transition occur in 70 wt.% Mn alloy between 80 °C and 95 °C (T_M_ = T_N_ = 95 °C). It is worth noting that the curves of the tanδ also exhibit transitions at the same temperatures. The tanδ value decreases sharply after T_M_, which means that the damping capacity of the alloy decreases significantly following the paramagnetic transition. These observations are consistent with those reported by Masumoto et al. [27], Wang et al. [28] and Yin et al. [14]. Both T_M_ and T_N_ increase with increasing Mn content. The Mn element is an austenite-stabilizing element. When the Mn content increases, the fcc phase in the Mn-Cu alloy is more stable, and more thermal activation energy is needed to complete the martensitic transformation, which consequently raises T_M_. The spinodal decomposition in the alloy with higher Mn content forms more Mn-rich regions during aging [29]. The stronger magnetic interaction between Mn atoms in the Mn-rich region helps to stabilize the antiferromagnetic ordered state [30], so the higher Mn alloy requires more thermal energy to undergo the paramagnetic transition, which makes the T_N_ increase. The higher the T_N_ is, the wider the temperature range in which the alloy maintains the antiferromagnetic state, resulting in superior damping properties within this range. This is particularly important for achieving good damping properties in a wider temperature range or at higher temperatures.

In all Mn-Cu alloys, tanδ initially increases with temperature until it reaches a maximum value at a certain temperature, defined as T_peak_. For the 70 wt.% Mn alloy, the maximum tanδ is approximately 0.064, occurring at T_peak_ = 5 °C. The maximum tanδ of the 75 wt.% Mn alloy decreases to approximately 0.05, and T_peak_ shifts down to 0 °C. With the 80 wt.% Mn alloy, the maximum tanδ further decreases slightly to approximately 0.046 and T_peak_ reaches −10 °C. In summary, as the Mn content increases, both the peak tanδ value and T_peak_ continuously decrease. This trend is likely strongly correlated with the phase constitution and microstructure. tanδ can still maintain a high level near the T_M_, and an inconspicuous subpeak is observed in the tanδ curves of 75 wt.% Mn and 80 wt.% Mn alloys near the T_M_, which may be caused by the martensitic transformation. Since T_M_ with T_N_ for the 70 wt.% Mn alloy coincides, no subpeak is evident in its tanδ curve. Above T_M_, as the paramagnetic transition begins, the tanδ value drops sharply to a very low level.

**Figure 9:**
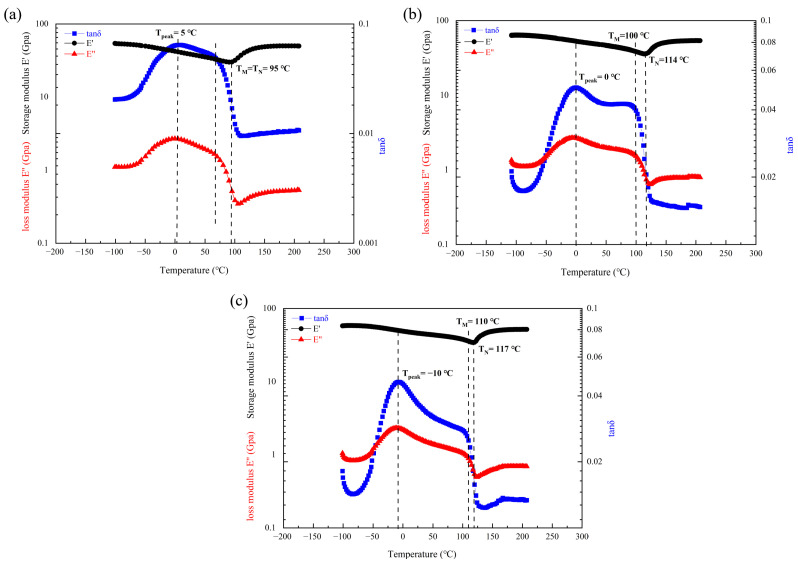
Temperature dependence of storage modulus (E′) and loss modulus (E′′) for Mn-Cu alloys with different Mn contents. (**a**) 70 wt.% Mn; (**b**) 75 wt.% Mn; (**c**) 80 wt.% Mn.

To further clarify the effect of Mn content on the damping mechanism, the storage modulus (E′) and loss modulus (E″) of the three alloys were analyzed as a function of temperature, as shown in Figure 9. The variation in the loss modulus E″ with temperature coincides exactly with that of tanδ, with the same peak temperature: approximately 5 °C (2.7 GPa) for the 70 wt.% Mn alloy, 0 °C (2.68 GPa) for the 75 wt.% Mn alloy, and −10 °C (2.3 GPa) for the 80 wt.% Mn alloy. The peak value of E” decreases with increasing Mn content, indicating that the lower Mn alloy exhibits a higher energy dissipation capacity. The storage modulus E′ of all three alloys gradually decreases to a minimum over a wide temperature range before increasing again. The minimum E′ values are reached at 95 °C (30 GPa) for the 70 wt.% Mn alloy, 114 °C (35.9 GPa) for the 75 wt.% Mn alloy, and 117 °C (35 GPa) for the 80 wt.% Mn alloy. The temperature at the minimum E′ is the paramagnetic transition temperature T_N_ [14], which is consistent with T_N_ inferred from the change of α_phys in Figure 8. Near the T_peak_ of the Mn-Cu alloys, the storage modulus E′ shows a slow and continuous decrease without any abrupt change or plateau. This behavior may result from the reversible slip of the twin boundaries under the synergistic effect of thermal activation energy and external alternating stress, resulting in elastic softening of the alloys. This behavior is a typical mechanical response of the twin relaxation process, indicating that the energy dissipation is mainly due to the viscous motion of the mobile twin boundaries rather than to phase transformation. The storage modulus E′ does not change significantly in near T_M_, likely because martensitic transformation primarily occurred during the aging treatment of the alloy, and only a small amount of additional transformation takes place during the DMA heating process, so the small amount of elastic softening caused by martensitic transformation does not cause a large fluctuation of E′. E′ exhibits a significant turning point at T_N_, changing from a continuous decline to a gradual increase and finally leveling off. This behavior is a direct consequence of the completion of the paramagnetic transition. When the temperature increases beyond T_N_ into the paramagnetic state, the antiferromagnetic order is disrupted, and the magneto-elastic coupling effect disappears. The originally softened lattice recovers its intrinsic stiffness, so the storage modulus E′ to rise. Concurrently, tanδ decreases sharply above T_N_. The reason for this sharp decrease is that, following the paramagnetic transition, the destruction of the antiferromagnetic order and the disappearance of the magneto-elastic coupling deprive the twin boundaries of the elastic driving forces necessary for their reversible motion. As a result, their mobility is severely limited, which prevents the effective dissipation of vibrational energy, leading to a sharp deterioration in the damping performance.

In conclusion, although the tanδ values and characteristic temperatures (T_peak_, T_M_ and T_N_) vary slightly with Mn content, the damping mechanisms of the three alloys are consistent. The main peak of the tanδ curve originates from the relaxation motion of the high-density twin boundaries (observed in Figure 3 and Figure 4) produced by aging, the less obvious secondary peak is derived from the martensitic transformation, and the damping performance decreases sharply after the paramagnetic transition. The differences in damping properties among alloys with varying Mn contents arise from their microstructure. Large numbers of twin structures were observed in all alloys, and multiple sets of intersecting twin bands were present within the small grains (Figure 3d, f), while the twinned bands within the large grains were straight and arranged as long, continuous strips extending through the grains (Figure 3b). The area-weighted average grain size of the 70 wt.% Mn alloy was 140 μm, whereas those of the 75 wt.% Mn and 80 wt.% Mn alloys were 26 μm and 32 μm respectively. Clearly, the 70 wt.% Mn alloy contained more long, continuous twin boundaries, while the twin boundaries in the 75 wt.% Mn and 80 wt.% Mn alloys were mostly short and intersecting. Studies [29,30] have shown that although multiple intersecting twin structures contain a higher density of twin boundaries, a large number of twin boundaries will be pinned near the intersection point. That is, each twin boundary will be divided into short fragments by multiple nodes, and the degree of freedom of movement is severely limited, which requires a higher activation energy for movement, and the damping performance of the alloy will decrease. In contrast, regular, straight twin boundaries can move freely over larger distances, thus improving the damping performance. This is the primary reason for the observed decrease in damping capacity with increasing Mn content. For the 80 wt.% Mn alloy, although its grain size is slightly larger than that of the 75 wt.% Mn alloy, more α-Mn particles with larger sizes precipitate during the aging process (Figure 4), along with a greater number of grains oriented along the [111] direction (Figure 2), resulting in a decrease in its damping properties.

## 4. Discussion

In this study, the XRD patterns (Figure 1) of the solution-treated and aged Mn-Cu alloys exhibited splitting of the fcc-MnCu (111) and (200) peaks into fct-MnCu peaks. This indicates that the martensitic transformation occurred during cooling following the solution and aging treatment, resulting in the formation of a twinned microstructure, as observed by SEM and TEM. Combined with the results shown in Figure 2 and Figure 3, grain refinement occurred with increasing Mn content under the same heat treatment regime. However, this refinement effect diminished when the Mn content exceeded 75 wt.%. After solution and aging treatment, smaller grains contained a higher number of twins, more twin variants, and more intersecting twin structures. In contrast, larger grains exhibited fewer twins with larger inter-twin spacing, fewer twin variants, and predominantly regular, straight twin bands with almost no intersecting twin structures. This is consistent with the results reported by Li et al. [8] and Yan et al. [31]. Additionally, the number and size of α-Mn particles at the grain boundaries increase with the increase in Mn content.

These microstructural discrepancies in turn led to variations in mechanical properties and damping capacity. The 75 wt.% Mn alloy exhibited the finest grain size, consisting predominantly of small grains, which resulted in the largest number of twins and intersecting twin structures. Grain boundaries, twin boundaries, and intersecting twin structures impeded dislocation motion and coordinated grain deformation, thus enabling the 75 wt.% Mn alloy to achieve the greatest tensile strength while maintaining relatively high elongation. Cracks in the 75 wt.% Mn alloy were observed to originate from the fragmentation of small grains, as revealed by in situ SEM observations and fracture surface analysis. These findings indicate that small grains played an important role in controlling deformation. With the tensile stress increased, unidirectional slip traces formed within the grains, and more grains were involved in deformation. When the maximum load was achieved, the grains displayed multidirectional slip traces (slip steps), which triggered shear failure and the formation of transgranular cracks. Finally, these crack sources led to fracture.

Under the present experimental conditions, the physical expansion coefficient curve of the Mn-Cu alloy has no correlation with the primary tanδ peak, but is related to the secondary peak. Based on the experimental data (Figure 8 and Figure 9), it can be inferred that the secondary peak is caused by the martensitic transformation. As the content of Mn increases, the coupling between the martensitic transformation and the paramagnetic transition weakens, and the characteristic temperatures of both martensitic transformation and paramagnetic transition increase. This indicates that increasing the content of Mn raises the paramagnetic–antiferromagnetic transition temperature of the alloy, enabling it to maintain the antiferromagnetic state over a wider temperature range, and thus maintain higher damping performance. From the curves of tanδ, E′ and E′′ with temperature changes for alloys with different Mn contents (Figure 9), the peak value of tanδ originates from the relaxation motion of high-density twin boundaries generated during the aging and cooling process, rather than from the martensitic transformation. The same phenomenon was observed by Tian et al. [29] and Yin et al. [30]. Although intersecting twin structures in the grain can hinder and pin dislocations and improve the strength during deformation, it limits the relaxation motion of the twin boundaries. Therefore, although there are more twins in the grains of 75 wt.% Mn and 80 wt.% Mn alloys with fine grains, they also exhibit more intersecting twin structures, which results in a lower number of mobile twin boundaries compared to the 70 wt.% Mn alloy. This is the primary reason why the 70 wt.% Mn alloy exhibits the highest tanδ peak. In addition, with the increase in Mn content, both the number and size of α-Mn particles at the grain boundaries are increased, which also contributes to the decrease in damping performance. In summary, the damping mechanism of Mn-Cu alloys does not vary with Mn content, which is dominated by twin boundary relaxation motion, supplemented by martensitic transformation and paramagnetic transition.

## 5. Conclusions

In this work, the influence of compositional variations in the quaternary Mn-Cu-Ni-Fe system, specifically changes in Mn content, on the microstructure, mechanical properties, and damping capacity of the alloys was systematically investigated. Under the present experimental conditions, the following conclusions can be drawn:

(1) Microstructure: In the range of Mn concentration studied (70–80 wt.%), Mn-Cu alloys were found to consist of fcc and fct phases after aging, with a small amount of α-Mn phase detected at the grain boundaries. The increase of manganese content leads to significant grain refinement, but this refinement weakens when the Mn content exceeds 75 wt.%. The 70 wt.% Mn alloy has a mixed-grain structure (average area-weighted grain size of 140 μm) with long, straight twin bands. The average area-weighted grain sizes of the 75 wt.% Mn and 80 wt.% Mn alloys are 26 μm and 32 μm, respectively, and contain numerous intersecting twin bands.
(2) Mechanical properties: The effect of Mn content on the mechanical properties is significant. Both tensile and yield strength first increase and then decline when increasing Mn content, with them reaching their maximum values at 75 wt.% Mn (UTS = 534 MPa, YS = 263 MPa). Elongation shows a slight decreasing trend. The 75 wt.% Mn alloy has the best combination of strength and ductility owing to its fine, uniform grain structure and the presence of intersecting twin bands.
(3) Damping capacity: With increasing Mn content, the martensitic transformation temperature and paramagnetic transition temperature increase, thus broadening the antiferromagnetic temperature range. With the increase in Mn content, the peak value and T_peak_ of tanδ decreased, and the 70 wt.% Mn alloy exhibiting the highest peak damping (tanδ = 0.064, T_peak_ = 5 °C). The damping behavior of all Mn-Cu alloys results from a composite process: the main peak is dominated by twin boundary relaxation, while the less obvious secondary peak corresponds to the martensitic transformation internal friction peak. After the paramagnetic transition, the damping performance decreases sharply.
(4) Application-oriented guidance: The 70 wt.% Mn alloy exhibits the highest damping capacity (tanδ = 0.064), whereas the 75 wt.% Mn alloy offers superior mechanical strength (UTS = 534 MPa). The optimal composition thus depends on the intended application: 70 wt.% Mn for high damping and 75 wt.% Mn for load-bearing applications. For high-temperature applications, increasing Mn content is a viable strategy.

## Figures and Tables

**Table 1:** Chemical compositions of the Mn-Cu alloys (wt.%).

Alloy	Nominal Mn	Mn	Cu	Ni	Fe
70 wt.% Mn	70	69.63 ± 0.03	23.08 ± 0.02	5.23 ± 0.04	2.06 ± 0.01
75 wt.% Mn	75	73.48 ± 0.05	20.40 ± 0.04	4.35 ± 0.03	1.77 ± 0.01
80 wt.% Mn	80	79.35 ± 0.04	15.71 ± 0.01	3.48 ± 0.04	1.46 ± 0.01

## Data Availability

The original contributions presented in this study are included in the article. Further inquiries can be directed to the corresponding author.
